# Serum biomarkers and non-invasive indices for discriminating MASLD among adults with dysglycaemia

**DOI:** 10.1515/med-2026-1556

**Published:** 2026-10-26

**Authors:** Kübranur Ünal, Leyla İbrahimkhanlı, Mehmet Emre Erol, Sabri Engin Altıntop, Nemat İbrahimkhanlı, Halit Nahit Şendur, Mahi Nur Cerit, Ethem Turgay Cerit

**Affiliations:** Department of Medical Biochemistry, Gazi University Faculty of Medicine, Ankara, Türkiye; Department of Medical Biochemistry, Institute of Health Sciences, Gazi University, Ankara, Türkiye; Department of Endocrinology and Metabolism, Faculty of Medicine, Gazi University, Ankara, Türkiye; Department of Radiology, Faculty of Medicine, Gazi University, Ankara, Türkiye

**Keywords:** dysglycaemia, body composition, Ultrasound-derived Hepatic Fat Fraction (USFF), visceral adiposity

## Abstract

**Objectives:**

Metabolic dysfunction–associated steatotic liver disease (MASLD) is the most prevalent chronic liver disease worldwide. Although dysglycaemia is a major risk factor, additional cardiometabolic disturbances contribute to the development of MASLD. This study aimed to identify parameters that distinguish MASLD among dysglycaemic individuals using body composition, anthropometric, biochemical, and imaging-based assessments.

**Methods:**

In this cross-sectional study, male and female adults were stratified into three groups: dysglycaemic individuals with MASLD (MASLD-Dys), dysglycaemic individuals without MASLD (Dys), and normoglycaemic individuals without MASLD as healthy controls. Imaging-based hepatic fat quantification, body composition, anthropometric and biochemical parameters, and non-invasive indices assessing hepatic steatosis and fibrosis were evaluated.

**Results:**

Compared with Dys participants, MASLD-Dys participants had higher body mass index (BMI), waist circumference, trunk fat mass, ultrasound-derived hepatic fat fraction (USFF), and Fatty Liver Index (FLI). The strongest between-group separation was observed for USFF (p<0.001). In receiver operating characteristic analysis, USFF showed the highest discriminative performance for MASLD-Dys vs. Dys, with an area under the curve of 0.925 and an optimal cut-off of ≥6.95 %.

**Conclusions:**

USFF was the strongest discriminator of MASLD among dysglycaemic adults, while FLI provided a practical non-imaging alternative. These findings support integrating hepatic fat quantification and body composition measures into MASLD risk stratification beyond glycaemic status alone.

## Introduction

Steatotic liver disease is an umbrella term for abnormal lipid accumulation within hepatocytes, with diverse aetiologies. In 2023, a multisociety Delphi consensus replaced the term non-alcoholic fatty liver disease (NAFLD) with metabolic dysfunction-associated steatotic liver disease (MASLD), emphasising the central role of cardiometabolic risk factors in hepatic steatosis [1]. Current European Association for the Study of the Liver–European Association for the Study of Diabetes–European Association for the Study of Obesity (EASL–EASD–EASO) guidelines define MASLD [2]. Recent meta-analytic data suggest that MASLD affects approximately 35 % of the adult population worldwide [3].

The clinical significance of MASLD lies not only in the presence of steatosis but also in its progressive nature. As the condition progresses over time, the presence of other metabolic risk factors, such as dysglycaemia, insulin resistance, and obesity, accelerates its progression. Dysglycaemia and T2DM are among the strongest metabolic factors associated with MASLD, but glycaemic status alone does not fully account for inter-individual differences in hepatic steatosis. Insulin resistance, adipose tissue dysfunction, ectopic lipid deposition, and atherogenic dyslipidaemia contribute jointly to the development and progression of MASLD in this population [4], [5], [6]. Therefore, dysglycaemia should be regarded as a major metabolic risk background rather than a sole determinant of MASLD. This nomenclatural transition represents more than a semantic change; it aims to provide a more precise definition of the disease, aligned with underlying pathobiology. The previous term, non-alcoholic fatty liver disease (NAFLD), has been criticised because it defines the condition by the absence of significant alcohol intake rather than by its dominant metabolic pathobiology. In addition, the term “fatty” has been considered potentially stigmatising and imprecise, and the heterogeneous terminology has contributed to challenges in biomarker development [7], [8], [9], [10]. The new nomenclature emphasises metabolic dysfunction as the central driver of disease and provides a more clinically useful framework for risk stratification and biomarker development [1].

The clinical burden of MASLD is largely driven by fibrosis progression, and fibrosis stage remains one of the strongest predictors of liver-related and extrahepatic outcomes [11]. In addition to serum-based scores, including the Fatty Liver Index (FLI), Fibrosis-4 Index (FIB-4), Aspartate Aminotransferase to Platelet Ratio Index (APRI), and NAFLD Fibrosis Score (NFS), non-invasive assessment has expanded to include the Enhanced Liver Fibrosis (ELF) score, which is calculated using serum levels of Procollagen Type III N-Terminal Propeptide (PIIINP), Hyaluronic Acid (HA), and Tissue Inhibitor of Metalloproteinase-1 (TIMP-1), which are key biomarkers of extracellular matrix remodelling [11], 12]. Imaging-based approaches, including the ultrasound-derived hepatic fat fraction (USFF), also provide quantitative assessment of hepatic fat content and complement serum-based tools for the non-invasive evaluation of steatotic liver disease [13], [14], [15]. In parallel, circulating biomarkers reflecting metabolic stress, inflammatory regulation, and extracellular matrix remodelling have gained interest as phenotyping tools for MASLD beyond conventional liver enzymes [16]. Serum 25-hydroxyvitamin D was also assessed as part of the broader metabolic phenotype, given reported associations of lower vitamin D levels with impaired insulin sensitivity, greater hepatic steatosis, and altered inflammatory regulation [17]. However, as non-invasive markers of inflammation, fibrogenesis, and metabolic stress gain clinical importance, their utility may vary considerably across dysglycaemic and other cardio-metabolic risk profiles. This variability exacerbates the challenges of heterogeneity in MASLD. Currently, there is a paucity of data regarding the performance of these metabolic stress markers in patients with dysglycaemia, particularly within the prediabetic population.

In addition to biochemical and score-based measurements, increasing evidence suggests that fat distribution plays a crucial role in determining the metabolic risk associated with MASLD, particularly in the context of dysglycaemia. Visceral and central fat accumulation, commonly assessed using anthropometric measures such as body mass index (BMI), waist circumference (WC), hip circumference (HC), waist-to-hip ratio (WHR), as well as body composition parameters including body fat mass (BFM) and trunk fat mass (TFM), is more closely linked to insulin resistance, hepatic lipid influx, and lipotoxicity than total adiposity alone [18]. Body composition analysis enables the non-invasive assessment of total and regional fat compartments, providing complementary information to conventional anthropometric indices. Previous studies suggest that individuals with dysglycaemia who preferentially accumulate fat in the central and trunk regions may be at an increased risk for hepatic steatosis and progressive MASLD, even in the absence of marked differences in glycaemic indices [5], 19]. In particular, recent CT-based evidence indicates that visceral fat area is more strongly associated with MASLD than body mass index, with sex-specific risk gradients that are not captured by conventional anthropometric indices alone [19]. Accordingly, integrating anthropometric measures and derived parameters with metabolic and hepatic markers has been proposed as a promising approach to improve phenotypic characterisation and risk stratification of MASLD in dysglycaemic populations [20].

Despite advances in MASLD nomenclature and non-invasive assessment, it remains unclear which metabolic, imaging-based, and body composition parameters best distinguish MASLD among dysglycaemic individuals. In this context, the present study was designed to investigate whether MASLD within a dysglycaemic background is characterised by a distinct phenotype associated with excess central adiposity and hepatic fat accumulation beyond glycaemic abnormality alone. Accordingly, this study aimed to identify the parameters that distinguish MASLD among dysglycaemic individuals by integrating biochemical markers, non-invasive scores, imaging-based quantification of hepatic fat, and detailed anthropometric and body composition assessments.

## Materials and methods

### Participant selection and classification

Participants were individuals aged 21−70 years with dysglycaemia (prediabetes or newly diagnosed type 2 diabetes mellitus (T2DM), not yet on an antidiabetic agent) and normoglycaemic volunteers presenting to the Endocrinology and Metabolic Diseases Outpatient Clinic of Gazi University, Faculty of Medicine Hospital.

Glycaemic status was classified according to the American Diabetes Association Standards of Care criteria [21]. Prediabetes was defined as fasting plasma glucose (FPG) of 100–125 mg/dL and/or glycated haemoglobin A1c (HbA1c) of 5.7–6.4 %; when available, a 2-hour plasma glucose value of 140–199 mg/dL during an oral glucose tolerance test (OGTT) was also considered diagnostic of prediabetes. Newly diagnosed T2DM was defined as FPG ≥126 mg/dL and/or HbA1c ≥6.5 %; when available, a 2-hour plasma glucose value ≥200 mg/dL during an OGTT was also considered diagnostic of diabetes. Glycaemic results were confirmed by repeat testing. HbA1c tests were performed in a standardised laboratory. In the present study, dysglycaemia was defined as the presence of either prediabetes or newly diagnosed, treatment-naïve T2DM. Participants receiving any antidiabetic treatment, including oral glucose-lowering agents, glucagon-like peptide-1 receptor agonists, or insulin, were excluded.

Healthy controls were defined as participants with FPG <100 mg/dL and HbA1c <5.7 %, without clinical evidence of metabolic dysfunction and without hepatic steatosis on ultrasound.

At the end of the stratification, the study population was divided into three groups: MASLD-Dys (dysglycaemic individuals with MASLD; n=49), Dys (dysglycaemic individuals without MASLD; n=11), and a normoglycaemic healthy control group without MASLD (n=17). The overall study design is illustrated in Figure 1, while the exclusion criteria are summarised in Table 1.

**Figure 1:**
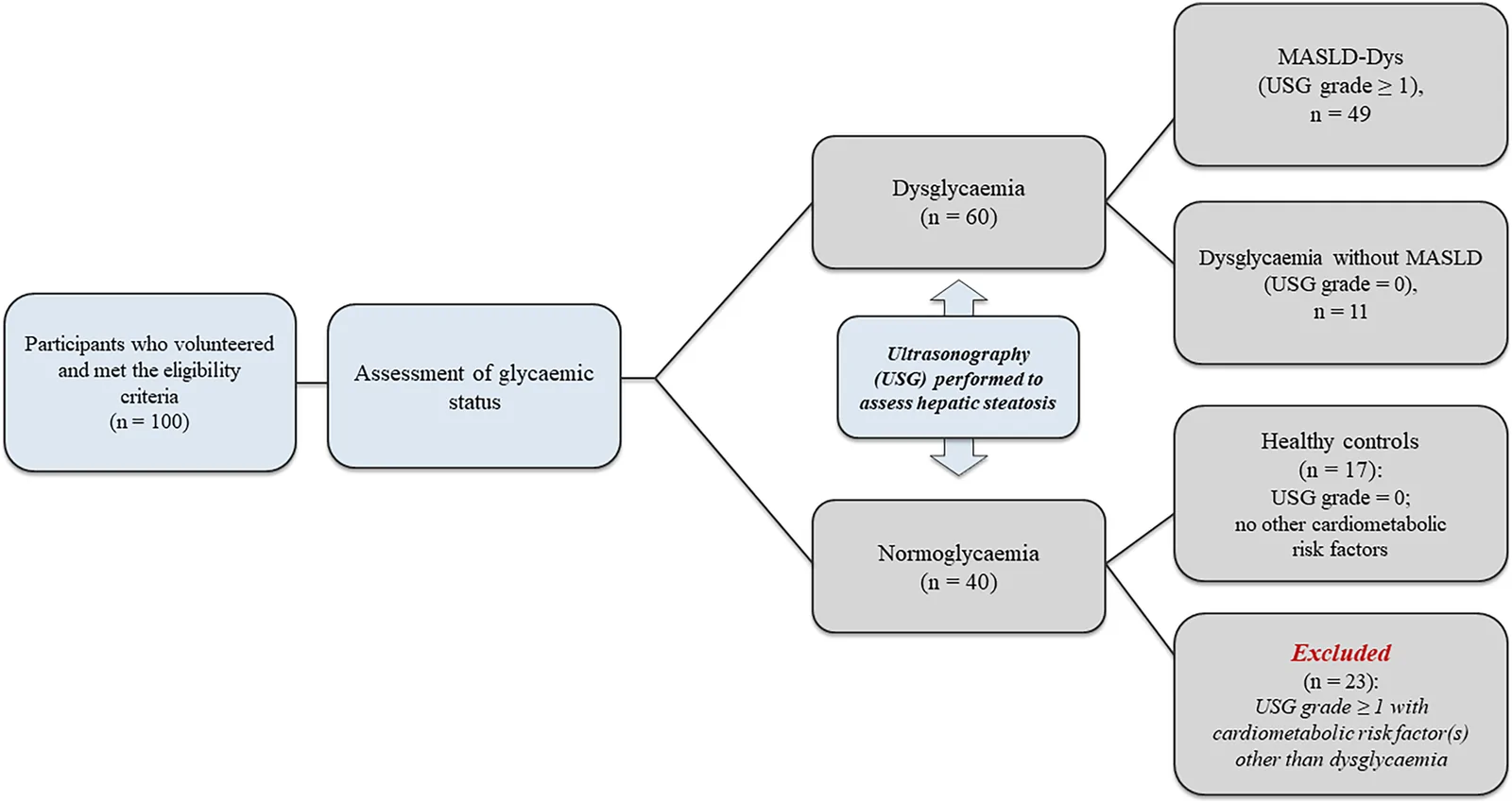
Flow diagram of participant selection and group stratification.

**Table 1:** Exclusion criteria applied in the study.

No	Exclusion criterion
i	Severe psychiatric or intellectual disorders impairing informed consent or compliance
ii	Presence of active malignancy
iii	Alcohol consumption exceeding MASLD thresholds (≥30 g/day for men; ≥20 g/day for women) [22]
iv	Acute infection or chronic active infection with hepatitis B virus (HBV), hepatitis C virus (HCV), or human immunodeficiency virus (HIV).
v	Elevated C-reactive protein levels above the upper reference limit, indicating overt inflammatory or infectious conditions
vi	Current or prior use of antidiabetic, anti-inflammatory, or immunomodulatory medications that could interfere with metabolic or inflammation-related biomarkers

### Biochemical analysis

Blood samples were collected after at least 8 h of overnight fasting, and serum was separated by centrifugation and stored at −80 °C until analysis. Samples underwent a single freeze–thaw cycle and were not refrozen thereafter to minimise pre-analytical variability.

Fasting glucose (FG, mg/dL), serum albumin (g/dL), total cholesterol (TC, mg/dL), high-density lipoprotein cholesterol (HDL-C, mg/dL), low-density lipoprotein cholesterol (LDL-C, mg/dL; calculated only in samples with triglyceride levels <400 mg/dL), triglycerides (TG, mg/dL), aspartate aminotransferase (AST, U/L), alanine aminotransferase (ALT, U/L), and gamma-glutamyl transferase (GGT, U/L) were measured using Siemens Healthineers^®^ commercial kits on the Siemens ADVIA Chemistry XPT auto-analyser via photometric methods.

Platelet count ( × 10^9^/L) was obtained using the Beckman Coulter^®^ DxH 900 haematology analyser. Fasting insulin (FI, µIU/mL) and 25-hydroxy vitamin D (25-OH D; ng/mL) concentrations were measured by chemiluminescent immunoassay (CLIA) using the Siemens ADVIA Centaur XPT hormone analyser. Levels of HbA1c were determined using ion-exchange high-performance liquid chromatography (HPLC) on a Tosoh G8 instrument. The TG/HDL-C ratio was calculated as the ratio of serum triglycerides to HDL-C [23]. The HOMA-IR and HOMA-β indices were calculated from FG and FI values using the Homeostatic Model Assessment method described by Matthews et al. [24].

Serum FGF-21, TIMP-1, and PIIINP concentrations were measured using commercially available quantitative sandwich enzyme-linked immunosorbent assay (ELISA) kits from Elabscience (Catalog Nos. E-EL-H0074, E-EL-H0184, and E-EL-H0183, respectively; Elabscience Biotechnology Co., Ltd., Wuhan, China). Serum hyaluronic acid (HA) was measured using a Cusabio ELISA kit (Catalog No. CSB-E04805h; Cusabio Biotech Co., Ltd., Wuhan, China). The analytical sensitivities were 18.75 pg/mL for FGF-21, 0.09 ng/mL for TIMP-1, 14.06 pg/mL for PIIINP, and 0.039 ng/mL for HA. The corresponding detection ranges were 31.25–2,000 pg/mL, 0.16–10 ng/mL, 23.44–1,500 pg/mL, and 0.156–10 ng/mL, respectively. Serum TIMP-1 and PIIINP samples were diluted 1:20 with sample dilution buffer prior to analysis, and final serum concentrations were calculated by multiplying measured concentrations by the dilution factor. Values of PIIINP were converted from pg/mL to ng/mL for reporting. Assay precision characteristics, including intra- and inter-assay coefficients of variation, were within the manufacturer-specified analytical performance limits. Standard curves and sample concentrations were accepted according to the manufacturer’s validity criteria. All ELISA measurements were performed according to the manufacturer’s instructions.

### Body composition parameters and anthropometric measurements

Body composition parameters were determined using the TANITA BC-418MA segmental body composition analyser (Tanita Corporation, Tokyo, Japan) according to the manufacturer’s instructions. The parameters recorded included total body fat mass (BFM, kg), trunk fat mass (TFM, kg), and body mass index (BMI, kg/m^2^). Waist circumference (WC, cm) and hip circumference (HC, cm) were measured while the participant stood upright using a flexible, non-stretch tape measure. WC was recorded at the narrowest point of the waist, and HC at the widest point of the hips. The waist-to-hip ratio (WHR) was calculated as WC divided by HC [25]. To ensure inter-measurement consistency, all body composition and anthropometric assessments were performed by the same trained researcher following a standardised protocol, and all measurements were entered into the study database immediately after collection.

### Calculation of non-invasive scores and metabolic indices

Non-invasive fibrosis and steatosis scores were calculated using established and previously validated formulas. The Fatty Liver Index (FLI) was calculated using BMI, WC, TG, and GGT levels, as defined by Bedogni et al. [26]. The NFS was calculated using the model proposed by Angulo et al., incorporating age, BMI, glycaemic status, platelet count, albumin level, and the AST/ALT ratio [27]. The FIB-4 index was calculated using the formula introduced by Sterling et al., which incorporates age, AST, ALT, and platelet count [28]. The APRI was calculated using the method initially described by Wai et al. [29]. The ELF score was calculated as originally described by Rosenberg et al., based on serum concentrations of HA, PIIINP, and TIMP-1 [30].

### Ultrasonographic hepatic examination

Hepatic steatosis grades, as determined by abdominal ultrasonography performed in the Radiology Department, were recorded in the study database. All participants underwent hepatic ultrasonography using an RS85 Prestige ultrasound system (Samsung Medison Co., Ltd., Seoul, South Korea) with a 1–7 MHz convex probe. Examinations were performed in the supine position after at least 6 h of fasting, and hepatic fat content was qualitatively measured via the right intercostal approach. On B-mode ultrasonography, hepatic steatosis was visually graded on a 0–3 scale (0=none). Ultrasound-derived hepatic fat fraction (USFF) was quantified using Tissue Attenuation Imaging (TAI) and Tissue Scatter Distribution Imaging (TSI), proprietary imaging modalities integrated into the RS85 Prestige ultrasound system (Samsung Medison Co., Ltd., Seoul, South Korea). Based on combined TAI and TSI measurements, the ultrasound-derived hepatic fat fraction (USFF, %) was automatically calculated by the device software and recorded for further analysis [31].

### Statistical analysis

Statistical analyses were performed using IBM SPSS Statistics version 25.0 (IBM Corp., Armonk, NY, USA). Python version 3.10 (Python Software Foundation, Wilmington, DE, USA) was used to construct the correlation matrix and perform multiple-testing adjustment for the correlation analyses using the pandas, SciPy, statsmodels, and matplotlib libraries. Group-comparison graphs were generated using GraphPad Prism (GraphPad Software, Boston, MA, USA).

The normality of each continuous variable was assessed within each study subgroup using the Shapiro–Wilk test together with visual inspection of Q–Q plots. Homogeneity of variances across subgroups was evaluated using Levene’s test. A variable was considered normally distributed when the Shapiro–Wilk p-value exceeded 0.05 in all three study subgroups and the corresponding Q–Q plots showed no substantial deviation from normality; otherwise, non-parametric methods were applied. Continuous variables are presented as mean ± standard deviation (SD) for normally distributed variables and as median with interquartile range (IQR) for non-normally distributed variables. Categorical variables are presented as absolute frequencies and percentages.

For normally distributed continuous variables, one-way analysis of variance (ANOVA) was used as the omnibus test. When the homogeneity of variance assumption was satisfied, Tukey’s honestly significant difference (HSD) post hoc test was used for pairwise comparisons. When Levene’s test indicated significant heterogeneity of variances, Welch’s ANOVA was used, with the Games–Howell post-hoc procedure where appropriate. For non-normally distributed continuous variables, the Kruskal–Wallis H test was used as the omnibus test. When the overall Kruskal–Wallis test was significant, post hoc pairwise comparisons were performed with a Bonferroni adjustment for multiple comparisons. For categorical variables, between-group comparisons were performed using Pearson’s chi-square test or exact tests, as appropriate.

Bivariate associations were quantified using Spearman’s rank correlation coefficient (ρ). The correlation matrix presented in Figure 4 was restricted to the MASLD-Dys subgroup. To account for multiple testing across the correlation matrix, p-values were adjusted using the Benjamini–Hochberg false discovery rate (FDR) procedure.

To address potential sex-related effects, exploratory within-group sex comparisons were performed in the MASLD-Dys subgroup using Welch’s independent-samples t-test for normally distributed variables or the Mann–Whitney U test for non-normally distributed variables. This analysis was restricted to the MASLD-Dys subgroup because the small number of male participants in the Dys subgroup precluded reliable sex-stratified comparisons across all three study groups.

The diagnostic performance of selected parameters was evaluated using receiver operating characteristic (ROC) curve analysis. The area under the curve (AUC) and corresponding 95 % confidence intervals were calculated, and optimal cut-off values were determined using the Youden index. Sensitivity and specificity were calculated at the optimal cut-off. All statistical tests were two-tailed, and p<0.05 was considered statistically significant. Exact p-values were reported to three decimal places, with values below 0.001 reported as p<0.001.

### Research ethics and informed consent

This study was conducted at the Gazi University Faculty of Medicine Hospital in Ankara, Türkiye, between June and December 2025. The study protocol was carried out in accordance with the ethical principles of the Declaration of Helsinki. Ethical approval was obtained from the Gazi University Clinical Research Ethics Committee (Decision No. 095, May 16, 2025). The purpose, procedures, and potential risks of the study were explained in detail to all participants, and written informed consent was obtained from each participant prior to participation.

## Results

### Demographic and clinical characteristics

A total of 77 adult participants were included and stratified into three subgroups: 49 individuals with MASLD and dysglycaemia (MASLD-Dys; 28 females, 21 males), 11 individuals with dysglycaemia without hepatic steatosis (Dys; 8 females, 3 males), and 17 healthy controls (12 females, 5 males). The groups were comparable in terms of age and sex distribution (Table 2). Mean age was 49.4 ± 11.9 years in the MASLD-Dys group, 46.2 ± 14.3 years in the Dys group, and 43.0 ± 8.9 years in the healthy control group, with no statistically significant between-group difference. The distribution of female participants was also similar across the three subgroups (57.1 %, 72.7 %, and 70.6 %, respectively; p=0.458). Smoking, alcohol consumption, family history of diabetes, and hypertension did not differ significantly among the groups (all p>0.05).

**Table 2:** Demographic and clinical characteristics of the study groups.

Parameter	Control (n=17)	MASLD-dys (n=49)	Dys (n=11)	p-Value
Age, years	43.0 ± 8.9	49.4 ± 11.9	46.2 ± 14.3	0.147
Female sex, n (%)	12 (70.6 %)	28 (57.1 %)	8 (72.7 %)	0.458
Smoking, n (%)	6 (35.3 %)	15 (30.6 %)	5 (45.5 %)	0.635
Alcohol use, n (%)	5 (29.4 %)	6 (12.2 %)	1 (9.1 %)	0.194
Family history of DM, n (%)	6 (35.3 %)	31 (63.3 %)	6 (54.5 %)	0.134
Hypertension, n (%)	–	9 (18.4 %)	1 (9.1 %)	0.116
T2DM, n (%)	–	21 (42.9 %)	2 (18.2 %)	**0.003**
Prediabetes, n (%)	–	28 (57.1 %)	9 (81.8 %)	**<0.001**

Continuous data are presented as mean ± SD, and categorical data are presented as n (%). Age was compared using one-way ANOVA. Categorical variables were compared using Pearson’s chi-square test or exact tests, as appropriate. The p-values shown in the table represent overall between-group comparisons. Bold p-values indicate overall statistical significance (p<0.05).

### Biochemical and metabolic parameters

Biochemical and metabolic parameters are presented in Table 3.

**Table 3:** Biochemical and metabolic parameters of the study groups.

Parameter	Control (n=17)	MASLD-dys (n=49)	Dys (n=11)	p-Value
FG, mg/dL	84.0 (80.0–89.0)^b^	98.0 (87.0–117.0)^a^	90.0 (87.5–96.0)^a^	**<0.001**
FI, μU/mL	6.9 (4.8–8.6)^b^	11.4 (6.7–17.0)^a^	10.2 (6.9–12.2)^ab^	**0.009**
HbA1c, %	5.5 (5.2–5.6)^b^	6.3 (6.0–7.2)^a^	6.0 (5.9–6.3)^a^	**<0.001**
HOMA-IR	1.52 (1.01–1.90)^b^	3.20 (1.81–4.82)^a^	2.49 (1.51–3.56)^ab^	**0.001**
HOMA-β, %	115.3 (87.8–171.4)	102.4 (56.7–160.7)	106.0 (83.1–148.4)	0.644
C-peptide, ng/mL	2.07 (1.57–2.28)^b^	2.85 (2.24–3.83)^a^	2.50 (1.85–2.70)^ab^	**0.001**
TG, mg/dL	77.0 (63.0–99.0)^b^	146.0 (90.0–225.0)^a^	90.0 (71.0–115.0)^b^	**<0.001**
TC, mg/dL	188.2 ± 31.3	204.9 ± 44.7	182.4 ± 48.4	0.168
LDL-C, mg/dL	117.3 ± 26.0	128.3 ± 36.4	120.6 ± 41.4	0.494
VLDL-C, mg/dL	15.0 (13.0–20.0)^b^	29.0 (18.0–45.0)^a^	18.0 (14.0–23.0)^b^	**<0.001**
HDL-C, mg/dL	51.4 (48.1–64.2)^b^	41.8 (35.8–50.6)^a^	44.9 (37.5–48.2)^ab^	**0.005**
AST, U/L	19.0 (18.0–26.0)	22.0 (19.0–26.0)	18.0 (16.0–23.0)	0.120
ALT, U/L	18.0 (13.0–26.0)^ab^	28.0 (19.0–36.0)^a^	17.0 (14.0–21.5)^b^	**0.009**
GGT, U/L	12.0 (8.0–16.0)^b^	25.0 (17.0–33.0)^a^	21.0 (15.0–26.0)^ab^	**0.001**
ALP, U/L	60.0 (49.0–69.0)^b^	70.0 (58.0–89.0)^ab^	81.0 (67.5–97.0)^a^	**0.016**
Platelet count ( × 10^9^/L)	264 (213–298)	251 (212.5–322)	264 (237–297)	0.823
25-OH vitamin D, ng/mL	17.7 (9.6–21.7)	12.4 (8.5–21.0)	13.0 (5.8–16.2)	0.336

Values are presented as mean ± SD for normally distributed variables and median (IQR) for non-normally distributed variables, as determined by the Shapiro–Wilk test within each group. Normally distributed variables were compared using one-way ANOVA followed by Tukey’s HSD post hoc testing, where appropriate. Non-normally distributed variables were compared using the Kruskal–Wallis H test, followed by Bonferroni-adjusted post hoc pairwise comparisons when the overall test was significant. The p-values shown in the table represent overall between-group comparisons. Superscript letters indicate adjusted post-hoc pairwise comparisons within each row. Values labelled “a” and “b” differ significantly from each other, whereas values labelled “ab” do not differ significantly from either “a” or “b”. Bold p-values indicate overall statistical significance (p<0.05). MASLD-Dys, metabolic dysfunction-associated steatotic liver disease with dysglycaemia; Dys, dysglycaemic individuals without MASLD; FG, fasting glucose; FI, fasting insulin; HbA1c, glycated haemoglobin A1c; HOMA-IR, Homeostatic Model Assessment of Insulin Resistance; HOMA-β, Homeostatic Model Assessment of β-cell Function; TG, triglycerides; TC, total cholesterol; LDL-C, low-density lipoprotein cholesterol; VLDL-C, very-low-density lipoprotein cholesterol; HDL-C, high-density lipoprotein cholesterol; AST, aspartate aminotransferase; ALT, alanine aminotransferase; GGT, gamma-glutamyl transferase; ALP, alkaline phosphatase; 25-OH vitamin D, 25-hydroxyvitamin D.

Fasting glucose levels were 98.0 (87.0–117.0), 90.0 (87.5–96.0), and 84.0 (80.0–89.0) mg/dL in the MASLD-Dys, Dys, and healthy control groups, respectively (overall p<0.001). Levels were significantly higher in both MASLD-Dys and Dys than in healthy controls (adjusted p<0.001 and p=0.035, respectively). A similar pattern was observed for HbA1c [6.3 % (6.0–7.2), 6.0 % (5.9–6.3), and 5.5 % (5.2–5.6), respectively; overall p<0.001], with both dysglycaemic groups differing significantly from healthy controls (adjusted p<0.001 for both). Alkaline phosphatase levels also differed among groups [70.0 (58.0–89.0), 81.0 (67.5–97.0), and 60.0 (49.0–69.0) U/L; overall p=0.016], with higher values in Dys than in healthy controls (adjusted p=0.023) (Supplementary Figure 1).

Fasting insulin, HOMA-IR, and C-peptide differed significantly among groups (overall p=0.009, p=0.001, and p=0.001, respectively), with higher values in MASLD-Dys than in healthy controls (adjusted p=0.007, p=0.001, and p=0.001, respectively). No other pairwise comparisons were significant. No significant group difference was observed for HOMA-β [102.4 (56.7–160.7), 106.0 (83.1–148.4), and 115.3 (87.8–171.4), respectively; p=0.644].

Triglyceride and VLDL-C concentrations were higher in MASLD-Dys than in both Dys and healthy controls [TG: adjusted p=0.039 and p<0.001; VLDL-C: adjusted p=0.045 and p=0.001, respectively], with significant overall group differences for both variables (p<0.001). A significant group difference was also observed for HDL-C (overall p=0.005), with levels lower in MASLD-Dys than in healthy controls (adjusted p=0.005). Total cholesterol and LDL-C did not differ significantly among groups.

Among liver enzymes, ALT differed among groups (overall p=0.009) and was higher in MASLD-Dys than in Dys (adjusted p=0.034). A significant group difference was also observed for GGT (overall p=0.001), with higher levels in MASLD-Dys than in healthy controls (adjusted p=0.001). No significant between-group differences were observed for AST, serum 25-hydroxyvitamin D, and platelet count (p=0.120, p=0.336, and p=0.823, respectively).

### Anthropometric parameters, body composition, and non-invasive liver indices

Measures of hepatic fat content, anthropometry, and body composition differed significantly among groups (Table 4; Figure 2).

**Table 4:** Anthropometric parameters, body composition, serum biomarkers, and non-invasive liver indices of the study groups.

Parameter	Control (n=17)	MASLD-dys (n=49)	Dys (n=11)	p-Value
BMI, kg/m^2^	23.8 (21.0–26.9)^b^	29.2 (26.9–33.0)^a^	24.5 (22.8–29.7)^b^	**<0.001**
WC, cm	84.0 ± 10.6^b^	102.1 ± 13.4^a^	91.7 ± 13.4^b^	**<0.001**
HC, cm	101.0 (100.0–106.0)^b^	110.0 (103.0–116.0)^a^	103.0 (100.0–110.0)^ab^	**0.005**
WHR	0.79 (0.76–0.86)^b^	0.94 (0.85–0.98)^a^	0.87 (0.83–0.91)^ab^	**0.001**
BFM, kg	15.9 (13.7–21.8)^b^	27.4 (20.6–32.5)^a^	20.3 (15.1–23.4)^ab^	**0.001**
TFM, kg	9.5 ± 3.8^b^	14.5 ± 5.1^a^	9.9 ± 3.2^b^	**<0.001**
USFF, %	3.39 (1.00–4.44)^b^	11.61 (8.29–16.04)^a^	2.54 (1.07–5.26)^b^	**<0.001**
FLI	9.6 (5.0–20.9)^b^	63.2 (48.4–88.5)^a^	29.8 (18.2–49.2)^b^	**<0.001**
FIB-4	0.74 (0.49–0.97)	0.82 (0.60–1.08)	0.67 (0.52–1.18)	0.348
NFS	−3.42 ± 1.05^b^	−2.03 ± 1.23^a^	−2.77 ± 1.16^ab^	**<0.001**
APRI	0.23 (0.17–0.30)	0.24 (0.21–0.30)	0.21 (0.15–0.31)	0.444
FGF-21, pg/mL	88.3 (86.8–90.4)	88.9 (87.2–90.9)	88.9 (87.9–89.9)	0.601
TIMP-1, ng/mL	142.2 ± 19.1	156.4 ± 32.2	140.1 ± 36.5	0.095^c^
HA, ng/mL	354.6 (279.0–435.0)	439.6 (332.0–581.0)	389.5 (264.0–522.0)	0.076
PIIINP, ng/mL	0.67 (0.16–1.26)	1.24 (0.65–1.34)	1.25 (0.96–1.34)	0.090
ELF score	8.50 (7.57–9.14)^b^	9.11 (8.88–9.50)^a^	9.18 (8.79–9.55)^ab^	**0.010**

Values are presented as mean ± SD for normally distributed variables and median (IQR) for non-normally distributed variables, as determined by the Shapiro–Wilk test within each group. Normally distributed variables were compared using a one-way ANOVA followed by Tukey’s HSD post hoc testing. Welch’s ANOVA was used for TIMP-1 because Levene’s test indicated heteroscedasticity. Non-normally distributed variables were compared using the Kruskal–Wallis H test followed by Bonferroni-adjusted pairwise comparisons. The p-values shown in the table represent overall between-group comparisons. Superscript letters indicate adjusted post-hoc pairwise comparisons within each row. Values labelled “a” and “b” differ significantly from each other, whereas values labelled “ab” do not differ significantly from either “a” or “b”. Bold p-values indicate overall statistical significance (p<0.05). MASLD-Dys, metabolic dysfunction-associated steatotic liver disease with dysglycaemia; Dys, dysglycaemic individuals without MASLD; BMI, body mass index; WC, waist circumference; HC, hip circumference; WHR, waist-to-hip ratio; BFM, body fat mass; TFM, trunk fat mass; USFF, ultrasound-derived hepatic fat fraction; FLI, Fatty Liver Index; FIB-4, Fibrosis-4 Index; NFS, NAFLD Fibrosis Score; APRI, Aspartate Aminotransferase to Platelet Ratio Index; FGF-21, fibroblast growth factor 21; TIMP-1, tissue inhibitor of metalloproteinase-1; HA, hyaluronic acid; PIIINP, procollagen type III N-terminal propeptide; ELF, Enhanced Liver Fibrosis. ᶜThe p-value for TIMP-1 was calculated using Welch’s ANOVA because the assumption of homogeneity of variances was violated, as indicated by Levene’s test.

**Figure 2:**
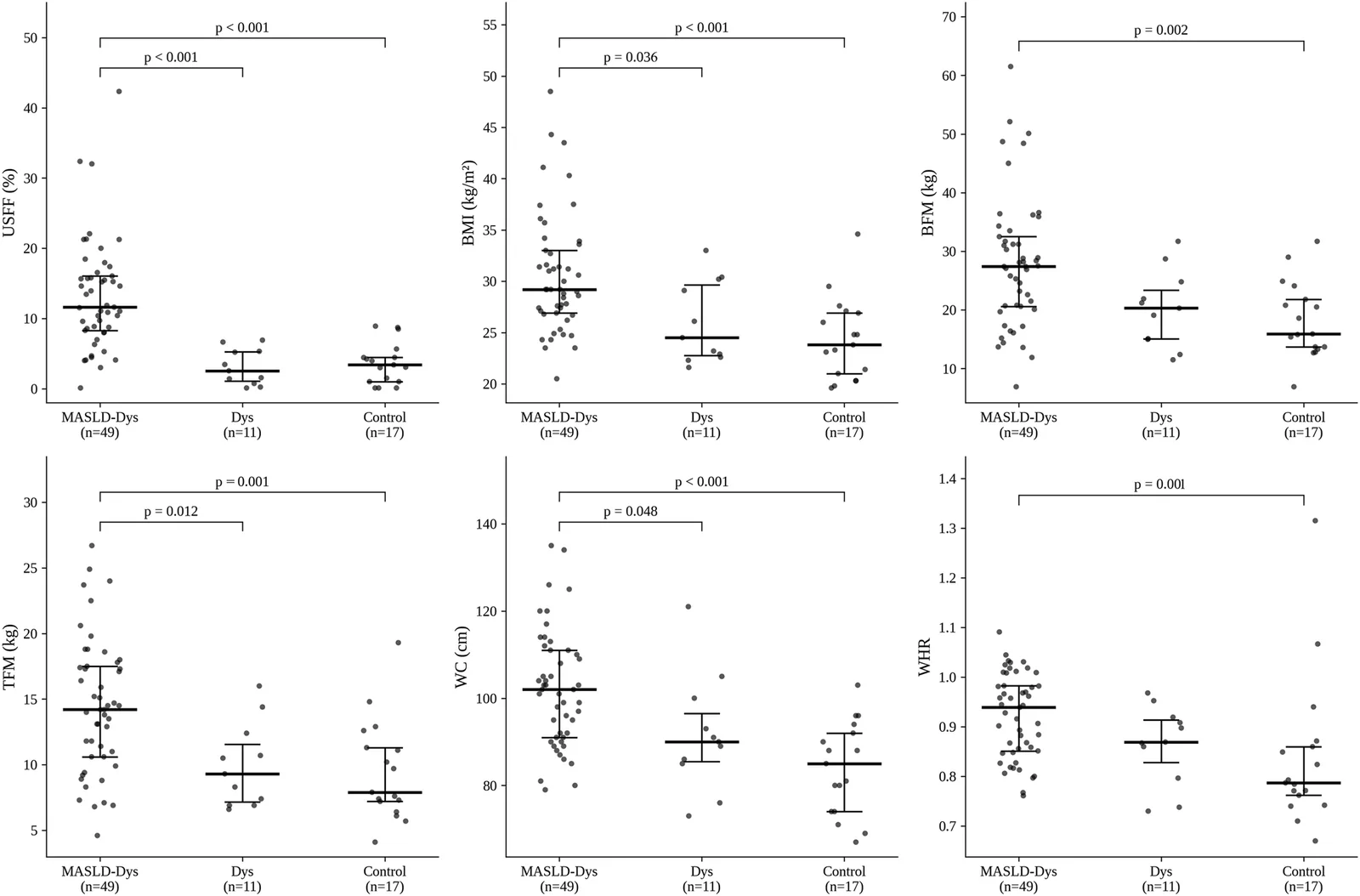
Group-wise comparison of hepatic fat content and anthropometric parameters. Data are displayed as individual values; horizontal bars indicate the median and error bars represent the interquartile range for visualisation. Inferential comparisons were performed after overall group testing using one-way ANOVA or the Kruskal–Wallis H test, followed by Tukey HSD or Bonferroni-adjusted pairwise comparisons, as appropriate based on the data distribution. Exact pairwise p-values are shown above the brackets, and only statistically significant comparisons are displayed. USFF, ultrasound-derived hepatic fat fraction; BMI, body mass index; BFM, body fat mass; TFM, trunk fat mass; WC, waist circumference; WHR, waist-to-hip ratio; MASLD-Dys, dysglycaemic individuals with metabolic dysfunction-associated steatotic liver disease; Dys, dysglycaemic individuals without MASLD.

For hepatic fat quantification, USFF values were 11.61 % (8.29–16.04), 2.54 % (1.07–5.26), and 3.39 % (1.00–4.44) in MASLD-Dys, Dys, and healthy controls, respectively (overall p<0.001). Values were higher in MASLD-Dys than in both comparison groups (adjusted p<0.001 for both).

Body mass index and waist circumference differed significantly among groups (overall p<0.001 for both). Values of BMI were higher in MASLD-Dys than in Dys and healthy controls (adjusted p=0.036 and p<0.001), while waist circumference was also greater in MASLD-Dys than in both groups (Tukey p=0.048 and p<0.001, respectively). Trunk fat mass showed the same pattern (overall p<0.001; Tukey p=0.012 and p=0.001, respectively).

Body fat mass, hip circumference, and waist-to-hip ratio also differed significantly among groups (overall p=0.001, p=0.005, and p=0.001, respectively), with significantly higher values in MASLD-Dys than in healthy controls (adjusted p=0.002, p=0.006, and p=0.001, respectively) (Figure 2).

Values of FLI differed significantly among groups (overall p<0.001) and were higher in MASLD-Dys than in Dys and healthy controls (adjusted p=0.024 and p<0.001, respectively). Serum TIMP-1, hyaluronic acid, PIIINP, and FGF-21 concentrations did not differ significantly among groups (p=0.095, p=0.076, p=0.090, and p=0.601, respectively). By contrast, FIB-4 and APRI were comparable among groups (p=0.348 and p=0.444).

The ELF score differed significantly among groups (overall p=0.010), with higher values in MASLD-Dys than in healthy controls (adjusted p=0.011). Values of NFS also differed significantly (overall p<0.001), with higher scores in MASLD-Dys than in healthy controls (Tukey p<0.001) (Figure 3).

**Figure 3:**
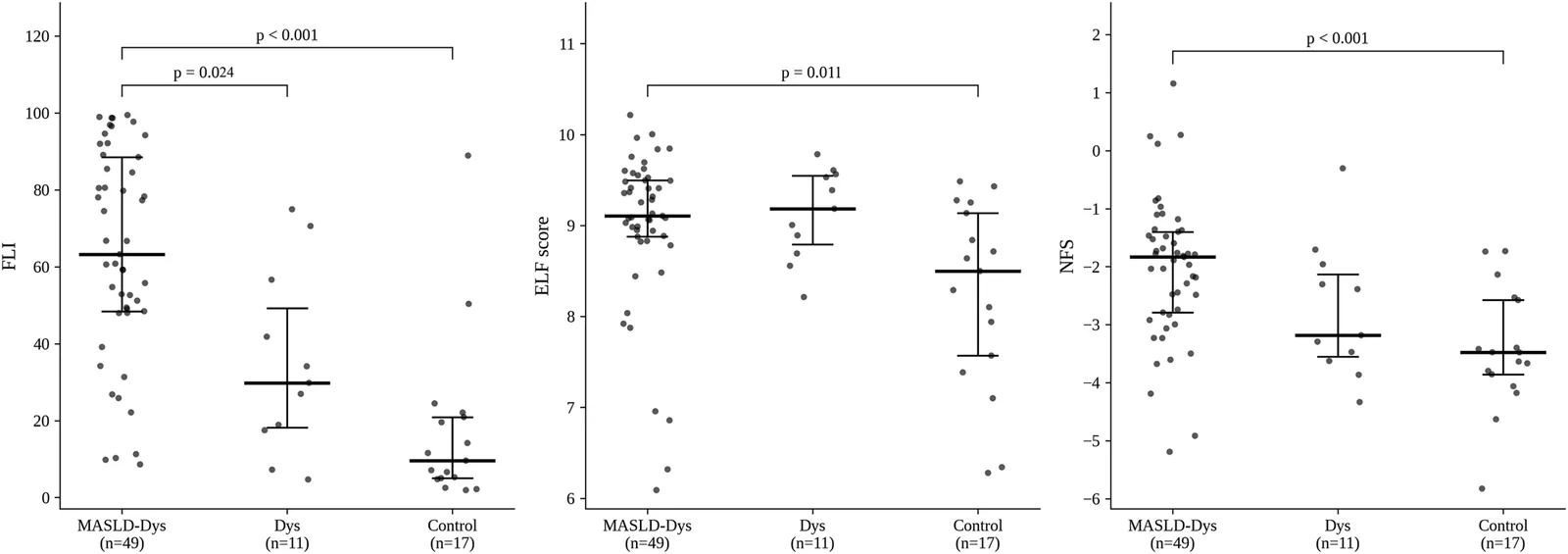
Group comparisons of liver-related non-invasive indices. Data are displayed as individual values; horizontal bars indicate the median and error bars represent the interquartile range for visualisation. Inferential comparisons were performed after overall group testing using one-way ANOVA or the Kruskal–Wallis H test, followed by Tukey HSD or Bonferroni-adjusted pairwise comparisons, as appropriate based on the data distribution. Exact pairwise p-values are shown above the brackets, and only statistically significant comparisons are displayed. FLI, Fatty Liver Index; ELF, Enhanced Liver Fibrosis score; NFS, NAFLD Fibrosis Score; MASLD-Dys, dysglycaemic individuals with metabolic dysfunction-associated steatotic liver disease; Dys, dysglycaemic individuals without MASLD.

### Correlation analysis

Correlation analysis restricted to the MASLD-Dys group (n=49) demonstrated positive associations between USFF and anthropometric and body-composition parameters, as well as FLI (Figure 4). Moderate positive correlations were observed between USFF and BFM (ρ=0.444), TFM (ρ=0.406), BMI (ρ=0.369), WC (ρ=0.342), HC (ρ=0.406), and FLI (ρ=0.369). Statistical significance in the correlation matrix was assessed using Benjamini–Hochberg false discovery rate (FDR)-adjusted p-values, as shown in Figure 4.

**Figure 4:**
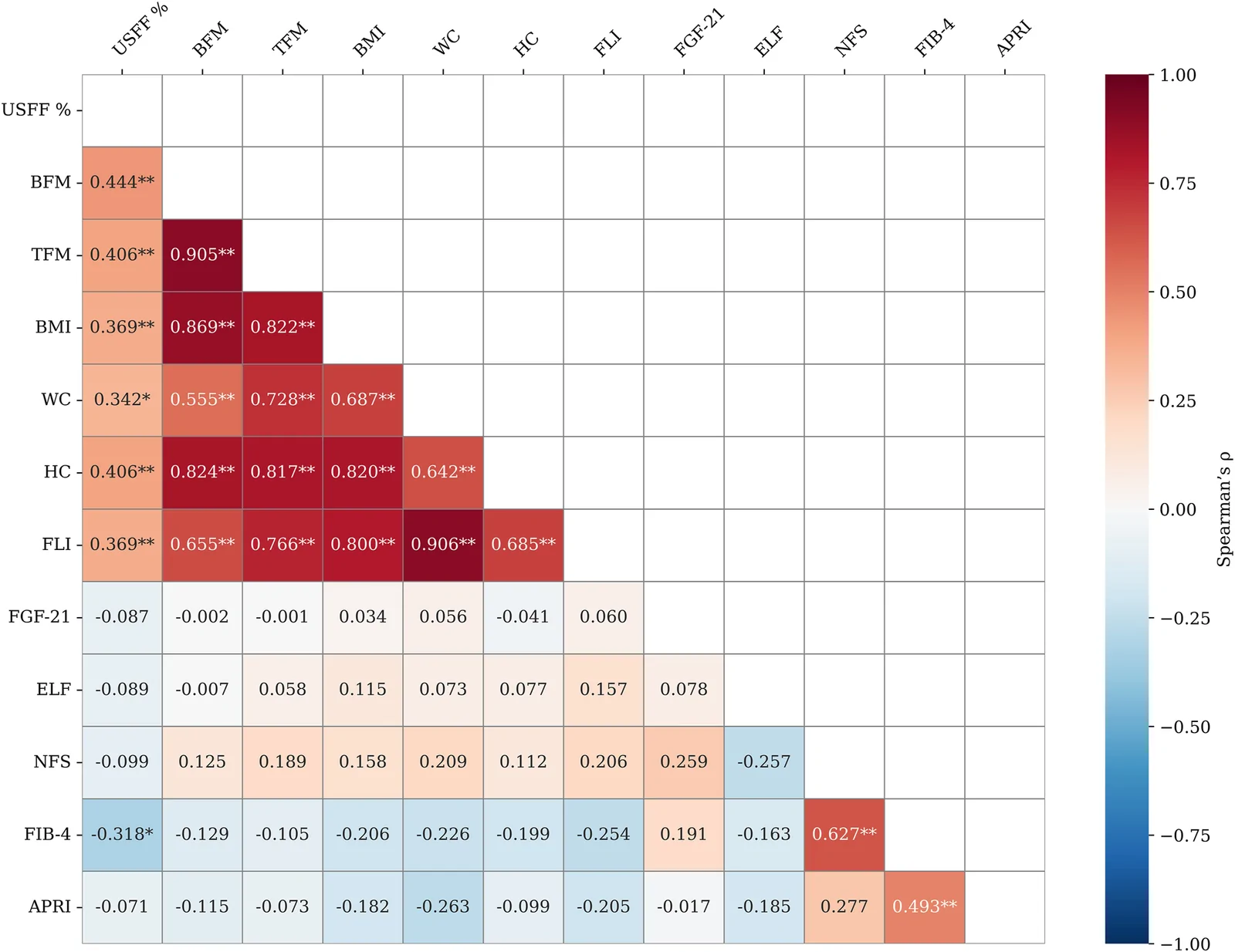
Correlation matrix between hepatic steatosis, body composition, and non-invasive indices in MASLD-Dys group. Correlation coefficients represent Spearman’s ρ. Statistical significance markers are based on Benjamini–Hochberg false discovery rate (FDR)-adjusted p-values: *FDR-adjusted p<0.05; **FDR-adjusted p<0.01. USFF, ultrasound-derived hepatic fat fraction; BFM, body fat mass; TFM, trunk fat mass; BMI, body mass index; WC, waist circumference; HC, hip circumference; HOMA-IR, Homeostatic Model Assessment of Insulin Resistance; TG, triglycerides; TG/HDL-C, triglyceride-to-HDL cholesterol ratio; FLI, Fatty Liver Index; FGF-21, fibroblast growth factor 21; ELF, Enhanced Liver Fibrosis score; NFS, NAFLD Fibrosis Score; FIB-4, Fibrosis-4 Index; APRI, Aspartate Aminotransferase to Platelet Ratio Index; MASLD-Dys, dysglycaemic individuals with metabolic dysfunction-associated steatotic liver disease.

The strongest associations with anthropometric and body-composition parameters were observed for FLI. Very strong correlations were observed between FLI and WC (ρ=0.906), BMI (ρ=0.800), and TFM (ρ=0.766), while strong correlations were observed with HC (ρ=0.685) and BFM (ρ=0.655). Strong correlations were also observed among the anthropometric and body-composition parameters themselves, indicating substantial interrelationships among measures of central and total adiposity.

Significant positive correlations were also observed between FIB-4 and other fibrosis-related indices, including NFS (ρ=0.627) and APRI (ρ=0.493), with significance assessed after FDR adjustment. In contrast, the ELF score and FGF-21 showed no significant correlations with USFF, FLI, or adiposity-related parameters in the correlation matrix.

In a separate exploratory analysis, Spearman correlations were used to examine associations between insulin resistance and lipid-related parameters (Supplementary Table S1). Within the MASLD-Dys group, HOMA-IR was positively correlated with triglycerides (ρ=0.505, p<0.001), VLDL-C (ρ=0.499, p<0.001), and the TG/HDL-C ratio (ρ=0.433, p=0.002).

### Sex-stratified analysis within the MASLD-Dys group

To address potential sex-related effects, an exploratory sex-stratified analysis was performed within the MASLD-Dys subgroup (Supplementary Table S2). Males showed significantly higher waist circumference, waist-to-hip ratio, FG, TG/HDL-C ratio, and GGT activity than females. Conversely, females showed significantly higher body fat mass, HDL-C, and HOMA-β than males. Importantly, USFF, FLI, BMI, trunk fat mass, HOMA-IR, and non-invasive fibrosis indices did not differ significantly between females and males. These findings suggest that although expected sex-related differences in body fat distribution and lipid-related metabolic profiles were present, the main hepatic fat signal in the MASLD-Dys subgroup was not explained primarily by sex.

### Diagnostic performance (ROC analysis)

Diagnostic performance was evaluated using ROC analysis of selected anthropometric, biochemical, body-composition, imaging-based, and composite parameters for discriminating MASLD-Dys from dysglycaemic individuals without MASLD (Table 5; Figure 5). Among all evaluated parameters, USFF demonstrated the highest diagnostic performance, with an AUC of 0.925. Acceptable discriminative performance was observed for FLI (AUC=0.787), TFM (AUC=0.773), TG (AUC=0.755), BMI (AUC=0.750), and ALT (AUC=0.742), whereas BFM and WC showed lower but still informative discrimination. Limited discriminative performance was observed for the ELF score, PIIINP, and FGF-21.

**Table 5:** Diagnostic performance of selected parameters for discriminating MASLD-Dys from dysglycaemic individuals without MASLD.

Parameter	AUC	Cut-off	Sens	Spec	p-Value	95 % CI	
						Lower bound	Upper bound
USFF, %	0.925	≥6.95	0.816	1.000	**<0.001**	0.857	0.993
FLI	0.787	≥44.88	0.796	0.727	**0.003**	0.650	0.924
TFM, kg	0.773	≥12.65	0.653	0.818	**0.005**	0.636	0.910
TG, mg/dL	0.755	≥136	0.551	1.000	**0.009**	0.630	0.880
BMI, kg/m^2^	0.750	≥26.15	0.816	0.636	**0.010**	0.580	0.921
ALT, U/L	0.742	≥22.50	0.633	0.818	**0.013**	0.570	0.914
BFM, kg	0.723	≥25.05	0.612	0.812	**0.022**	0.571	0.874
WC, cm	0.718	≥94.0	0.694	0.727	**0.025**	0.541	0.895

ROC curve analysis was performed for the clinically relevant discrimination scenario of MASLD-Dys versus dysglycaemic individuals without MASLD. AUC values are reported with 95 % confidence intervals. Optimal cut-off values were determined by maximising Youden’s J statistic. The bold values indicate statistically significant p-values (p<0.05) in the ROC curve analysis.

**Figure 5:**
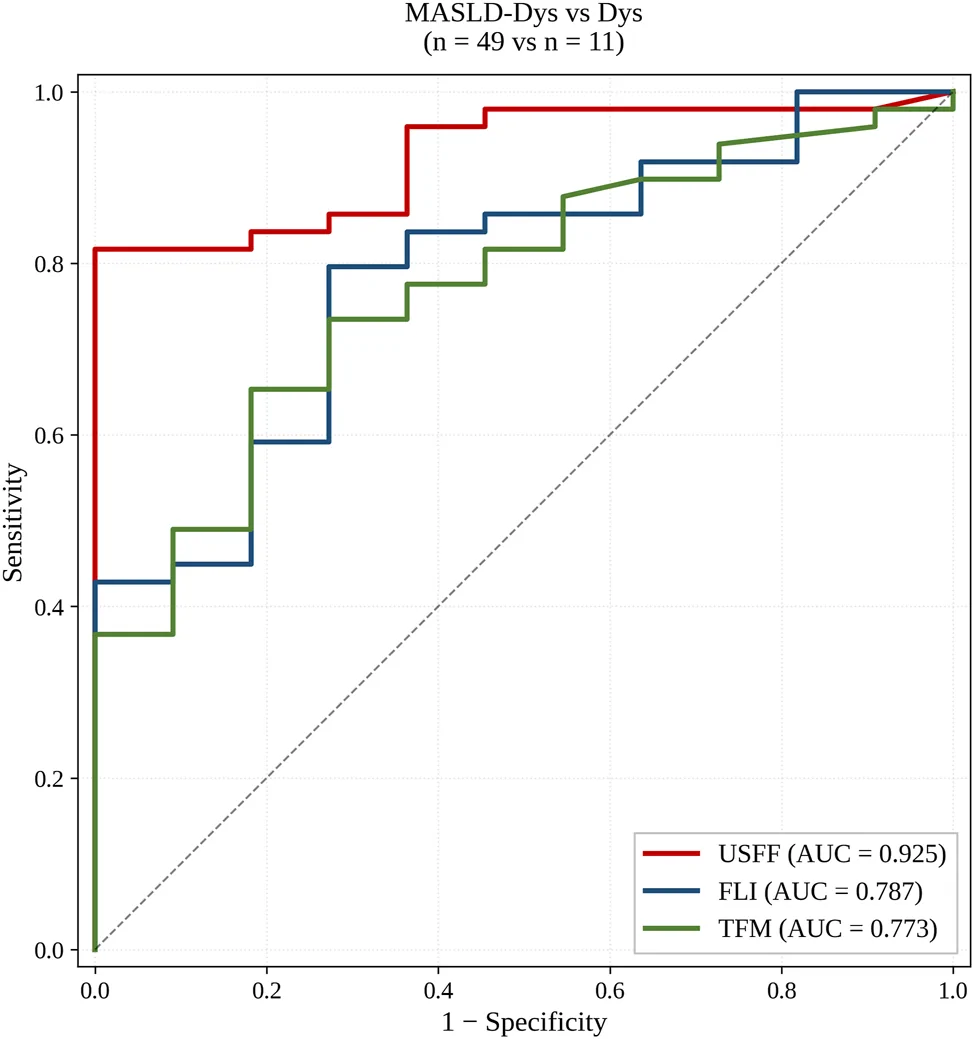
Diagnostic performance of non-invasive markers for MASLD-Dys group. The ROC curves show the diagnostic performance of selected parameters in MASLD-Dys vs. dysglycaemic individuals without MASLD. ROC, receiver operating characteristic; MASLD-Dys, dysglycaemic individuals with metabolic dysfunction-associated steatotic liver disease; AUC, area under the curve; USFF, ultrasound-derived hepatic fat fraction; FLI, Fatty Liver Index; TFM, trunk fat mass; TG, triglycerides; BMI, body mass index; ALT, alanine aminotransferase; BFM, body fat mass; WC, waist circumference.

At the optimal Youden-derived cut-off, USFF yielded 81.6 % sensitivity and 100 % specificity at a cut-off value of ≥6.95 %. At its optimal cut-off, FLI showed 79.6 % sensitivity and 72.7 % specificity at a cut-off value of ≥44.88, while TFM showed 65.3 % sensitivity and 81.8 % specificity at a cut-off value of ≥12.65 kg.

## Discussion

In this study, a MASLD group composed entirely of dysglycaemic individuals was compared with dysglycaemic individuals without MASLD and healthy controls to comprehensively evaluate biochemical, metabolic, and body composition markers that distinguish MASLD in the context of dysglycaemia.

This design enabled us to examine whether MASLD in dysglycaemic individuals reflects glycaemic abnormality alone or the combined effects of dysglycaemia with additional metabolic stressors. Furthermore, the inclusion of imaging-based, score-derived, biochemical, and body-composition variables enabled exploration of MASLD-related phenotypes from a multidimensional perspective, against the background of dysglycaemia. The data suggest that MASLD in a dysglycaemic background is not explained solely by the severity of glycaemic abnormality. Rather, hepatic lipid accumulation, excess adiposity, and central fat distribution appear to be key defining features of the MASLD-Dys phenotype.

In our cohort, MASLD was observed within a deliberately selected metabolically high-risk dysglycaemic background. This design allowed us to compare dysglycaemic individuals with and without MASLD and to identify factors beyond glycaemic status that distinguish the MASLD phenotype. This finding is consistent with previous observations that emphasised the high prevalence of MASLD among newly diagnosed T2DM patients [32]. Current consensus reports and guidelines highlight that individuals with prediabetes and T2DM are among the groups at highest risk of developing MASLD and emphasise the importance of systematic screening with non-invasive methods in this population [33]. Our findings support the clinical value of early risk assessment in these groups, including prediabetes, and demonstrate that dysglycaemia provides a strong predisposition to MASLD. However, our results clearly show that dysglycaemia alone is not sufficient to explain MASLD.

One of the key contributions of our study is the identification of factors that differentiate the presence of MASLD among individuals with dysglycaemia. When dysglycaemic individuals with MASLD were directly compared with dysglycaemic individuals without MASLD, parameters reflecting hepatic fat load, central/trunk adiposity, lipid-related metabolic burden, and liver enzyme activity, particularly USFF, FLI, BMI, WC, TFM, TG, VLDL-C, and ALT, differentiated the MASLD-Dys phenotype. In contrast, total body fat mass, hip circumference, and waist-to-hip ratio showed their clearest differences against healthy controls rather than consistently distinguishing MASLD-Dys from Dys. By comparison, glycaemic indices did not provide a significant distinction between the dysglycaemic groups. This finding supports our study hypothesis that, within a dysglycaemic background, MASLD is more closely associated with excess adiposity, hepatic fat accumulation, and lipid-related metabolic burden than with glycaemic abnormality alone. This interpretation is consistent with previous evidence highlighting the close relationships between visceral adiposity, adipose–liver crosstalk, and MASLD [20], 34]. These observations were further supported by correlation analyses within the MASLD-Dys group, in which USFF showed moderate correlations with anthropometric and body composition parameters, as well as with FLI. At the same time, FLI showed strong to very strong correlations with central and total adiposity measures, indicating that hepatic fat accumulation closely tracks overall and central adiposity in dysglycaemic MASLD.

Beyond glycaemic parameters, the distinction between MASLD and dysglycaemia in dysglycaemic individuals is characterised by a qualitative shift in metabolic load rather than simply a deterioration in glycaemic control. The observed pattern suggests an increased lipotoxic load and ectopic lipid accumulation, leading to hepatocellular stress, and indicates that dysglycaemia can serve as a permissive metabolic environment. In contrast, excessive adiposity and lipid redistribution appear to contribute more directly to the MASLD phenotype. Mechanistically, this pattern is compatible with an insulin resistance-driven atherogenic lipid phenotype. In the MASLD-Dys subgroup, HOMA-IR correlated with triglycerides, VLDL-C, and the TG/HDL-C ratio, but not with LDL-C, HDL-C, and TC, suggesting that insulin resistance in this cohort was more closely associated with triglyceride-rich lipoprotein metabolism than with LDL-C. This result may reflect increased adipose tissue lipolysis, enhanced hepatic free fatty acid influx, and increased hepatic VLDL production. The absence of significant between-group differences in HOMA-β further suggests that the MASLD-Dys phenotype was driven primarily by insulin resistance rather than overt β-cell failure. These findings support the close relationship between insulin resistance and dyslipidaemia, particularly when MASLD coexists with dysglycaemia.

In the present study, parameters reflecting central and trunk fat accumulation showed a more pronounced discriminatory pattern than glycaemic indices, supporting the notion that adiposity-related burden, rather than glycaemic severity per se, underlies the distinction of MASLD among dysglycaemic individuals. In the literature, obesity is an independent risk factor for MASLD, significantly increasing the risk of the disease [35]. However, the fact that MASLD can also be observed in non-obese individuals [36] and the high global prevalence of non-obese MASLD [37] emphasise the importance of considering the distribution and dynamic changes in fat accumulation, rather than relying solely on static measurements. Indeed, longitudinal studies have reported that the rate of BMI increase is strongly associated with the incidence of MASLD [38]. Our findings extend this concept by demonstrating that, in a dysglycaemic context, central and trunk fat accumulation is a key determinant distinguishing MASLD from dysglycaemia alone.

In the present study, USFF values were significantly higher in the MASLD-Dys group than in both the Dys and healthy control groups. Among the parameters evaluated in ROC analyses, USFF demonstrated the highest diagnostic accuracy, supporting its potential clinical utility for identifying and risk-stratifying MASLD in individuals with dysglycaemia. Previous studies have shown that USFF measurements are highly consistent with magnetic resonance imaging-proton density fat fraction (MRI-PDFF) and histopathological steatosis grades, providing strong diagnostic performance for the detection and quantification of hepatic steatosis in MASLD [13], [14], [15]. Taken together, our findings support the concept that USFF, as a non-invasive, reproducible, and quantitative measure, may be a promising tool for MASLD assessment, particularly in real-world risk-stratification settings. Consistent with this, USFF showed positive correlations with adiposity-related parameters (BMI, BFM, TFM, WC), indicating that hepatic fat fraction closely tracks overall and central fat burden [39].

The exploratory sex-stratified analysis further contextualised these findings in relation to known sex-related differences in body composition and MASLD risk. Previous body composition studies have shown that altered body composition in MASLD is closely linked to abdominal adiposity and hepatic steatosis [18], while recent computed tomography-based evidence indicates that visceral adiposity is strongly associated with MASLD in both sexes and may outperform BMI in sex-stratified models [19]. In our MASLD-Dys subgroup, males showed higher waist circumference, waist-to-hip ratio, FG, TG/HDL-C ratio, and GGT activity. In contrast, females showed higher HDL-C, body fat mass, and HOMA-β. Importantly, USFF, FLI, BMI, trunk fat mass, HOMA-IR, and non-invasive fibrosis indices did not differ significantly between sexes. These findings suggest that although expected sex-related differences in body fat distribution and metabolic phenotype were present, they did not primarily account for the hepatic fat accumulation observed in the MASLD-Dys subgroup. Nevertheless, because this analysis was exploratory and limited to a modest subgroup, the findings should be interpreted cautiously.

The biomarkers incorporated into the ELF score, including HA, TIMP-1, and PIIINP, are well-established markers of extracellular matrix remodelling, particularly in advanced fibrosis and steatohepatitis. In the present study, however, fibrosis-oriented biomarkers and scores showed limited diagnostic performance in ROC analyses, likely reflecting the predominantly early-stage, steatosis-dominant disease spectrum and the low prevalence of advanced fibrosis. Although TIMP-1 and HA levels were numerically higher in the MASLD-Dys group, these differences were not statistically significant, consistent with evidence that these markers primarily reflect advanced fibrotic burden rather than early steatosis [11], 40].

In contrast, the ELF score was significantly higher in the MASLD-Dys group than in healthy controls, whereas the Dys group did not differ significantly from either group. This pattern may reflect early extracellular matrix turnover or metabolic stress-related matrix remodelling rather than established fibrosis. However, the non-significant Dys-versus-control comparison limits interpretation of ELF as a dysglycaemia-related signal. Emerging data indicate that dysglycaemia and insulin resistance can trigger subtle fibrogenic signalling [12], 41].

Similarly, NFS values were significantly higher in the MASLD-Dys group compared with healthy controls, while no difference was observed between the MASLD-Dys and Dys groups. Given that NFS includes age, BMI, glucose status, and liver enzymes, this distribution may indicate overall metabolic activity rather than fibrotic differentiation. Conversely, both FIB-4 and APRI, which are more indicative of late fibrosis, did not differ between groups, further suggesting that this cohort is dominated by early disease. No significant group difference was observed for FGF-21. Although proposed as a marker of hepatic metabolic stress, FGF-21 levels exhibit significant variability. They may be more informative in advanced or progressive MASLD rather than in early, steatosis-predominant phenotypes, which may explain the lack of group differences observed in the present study [42], 43].

Among the evaluated non-invasive indices, FLI emerged as the most informative score in the present study. Strong correlations were observed between FLI and key anthropometric and body composition parameters reflecting central adiposity, including WC, BMI, TFM, and BFM, indicating a close association with an excess of centrally distributed fat. In ROC analyses, FLI demonstrated good diagnostic accuracy in discriminating MASLD-Dys individuals and ranked second overall, after USFF, among the evaluated non-invasive parameters. These findings align with previous studies, which indicate that FLI integrates both lipid parameters and adiposity-related measures, making it particularly sensitive for detecting steatosis rather than staging fibrosis. Hence, FLI may capture the metabolic–adiposity–driven component of MASLD more effectively than fibrosis-focused scores, supporting its utility as a practical tool for screening and risk stratification in dysglycaemic populations [44]. In contrast, fibrosis scores, such as FIB-4 and APRI, have limited ability to differentiate early MASLD because they primarily focus on advanced fibrosis [45], 46]. Indeed, these data indicate that in individuals with dysglycaemia, screening strategies should first target adiposity- and steatosis-focused tools, while reserving fibrosis scores to rule out advanced disease.

Serum 25-hydroxyvitamin D was included as part of the broader metabolic phenotype rather than as a primary diagnostic biomarker. Previous studies have linked vitamin D status to insulin sensitivity, hepatic lipid metabolism, inflammatory regulation, and fibrogenic pathways in MASLD-related metabolic dysfunction [17]. In the present cohort, however, 25-hydroxyvitamin D levels did not differ significantly among groups, despite low median values across all subgroups. This finding suggests that vitamin D did not provide MASLD-specific discriminatory value in our sample. One possible explanation is the high background prevalence of vitamin D insufficiency in the source population, which may have reduced between-group contrast and masked subtle MASLD-related differences. Therefore, our findings do not exclude a biological role for vitamin D in MASLD, but indicate that it was not useful for distinguishing MASLD within this dysglycaemic cohort.

A key strength of this study is the focused assessment of a dysglycaemic population, in which all MASLD cases were dysglycaemic, enabling clinically meaningful comparisons within a high-risk metabolic background. Inclusion of newly diagnosed and untreated individuals minimises the confounding effects of pharmacological interventions on metabolic and inflammatory parameters. Another significant strength of the study is its multidimensional assessment approach, which integrates imaging-derived parameters, non-invasive scores, biochemical markers, and detailed anthropometric and body composition measurements. This comprehensive strategy enabled identification of the underlying adiposity- and lipid-related mechanisms contributing to MASLD development in individuals with dysglycaemia.

This study has several limitations. First, the cross-sectional design precludes causal inference. Second, the single-centre setting and modest sample size, particularly in the Dys subgroup, limited statistical power and precluded reliable three-way sex-stratified comparisons. Therefore, subgroup-based findings, including ROC discrimination in the MASLD-Dys vs. Dys scenario, should be interpreted cautiously. Because of the modest sample size, particularly the small Dys subgroup, classification based on principal component analysis or multivariable prediction modelling was not performed to avoid overfitting and unstable model estimates. Larger prospective cohorts are required to validate whether combined biomarker, imaging, and body-composition signatures can improve MASLD classification within dysglycaemic populations. Third, hepatic steatosis was characterised non-invasively without liver biopsy or elastography-based fibrosis assessment, precluding definitive staging of steatohepatitis or fibrosis. Fourth, FGF-21 concentrations clustered within the lower portion of the assay standard curve, limiting discrimination of small inter-individual differences. Fifth, ELISA-based biomarker quantification was performed as a single measurement rather than in duplicate. Sixth, ROC-derived cut-offs were estimated within the same cohort and require external validation in independent prospective cohorts.

## Conclusions

In this cross-sectional study, USFF was the strongest discriminator of MASLD among dysglycaemic adults. The MASLD-Dys phenotype was characterised by central and trunk adiposity, hepatic fat accumulation, atherogenic dyslipidaemia, insulin resistance, and preserved HOMA-β. Fibrosis-oriented indices showed limited discriminatory ability, supporting an early, steatosis-dominant phenotype. These findings support a non-invasive evaluation framework integrating quantitative imaging, anthropometric, and metabolic measures for early MASLD risk stratification among dysglycaemic adults. The proposed cut-offs require external validation.

## Supplementary Material

Supplementary Material

Supplementary Material
