# Supplementary material for: Serum biomarkers and non-invasive indices for discriminating MASLD among adults with dysglycaemia
Source: Open Med (Wars). 2026 Oct 26;21(1):20261556. doi: 10.1515/med-2026-1556 (PMC13695451; doi:10.1515/med-2026-1556)
Supplement: Supplementary file 1 [file j_med-2026-1556_suppl_002.docx]

**Supplementary Table S1. Spearman correlations of HOMA-IR with lipid parameters**

| **Parameter** | **MASLD-Dys (n=49)** | **Dys (n=11)** | **Control (n=17)** |
| --- | --- | --- | --- |
| **TG, mg/dL** | **ρ = +0.505 (p < 0.001)** | ρ = +0.345 (p = 0.298) | ρ = +0.330 (p = 0.196) |
| **VLDL-C, mg/dL** | **ρ = +0.499 (p < 0.001)** | ρ = +0.330 (p = 0.322) | ρ = +0.325 (p = 0.203) |
| **TG/HDL-C ratio** | **ρ = +0.433 (p = 0.002)** | ρ = +0.209 (p = 0.537) | ρ = +0.324 (p = 0.205) |
| **HDL-C, mg/dL** | ρ = −0.107 (p = 0.465) | ρ = −0.127 (p = 0.709) | ρ = −0.120 (p = 0.645) |
| **LDL-C, mg/dL** | ρ = +0.139 (p = 0.342) | ρ = −0.182 (p = 0.593) | ρ = +0.208 (p = 0.422) |
| **TC, mg/dL** | ρ = +0.183 (p = 0.207) | ρ = −0.182 (p = 0.593) | ρ = +0.232 (p = 0.371) |

Spearman’s rank correlation coefficients (ρ) are shown with corresponding unadjusted p-values. These analyses were exploratory and were not adjusted for multiple comparisons. Bold values indicate nominal statistical significance (p < 0.05). MASLD-Dys, metabolic dysfunction-associated steatotic liver disease with dysglycaemia; Dys, dysglycaemic individuals without MASLD; Control, healthy controls; HOMA-IR, Homeostatic Model Assessment of Insulin Resistance; TG, triglycerides; VLDL-C, very-low-density lipoprotein cholesterol; HDL-C, high-density lipoprotein cholesterol; LDL-C, low-density lipoprotein cholesterol; TC, total cholesterol.

**Supplementary Table S2. Sex-stratified analysis within the MASLD-Dys subgroup**

| **Parameter** | **Females (n=28)** | **Males (n=21)** | **p** |
| --- | --- | --- | --- |
| **Age, years** | 48.1 ± 11.4 | 51.1 ± 12.7 | 0.399 |
| **BMI, kg/m²** | 30.5 (27.1–34.5) | 28.4 (26.8–31.2) | 0.169 |
| **WC, cm** | 96.0 (89.0–106.0) | 104.0 (99.0–112.0) | **0.034** |
| **HC, cm** | 111.5 (106.8–119.8) | 108.0 (102.0–112.0) | 0.060 |
| **WHR** | 0.86 (0.82–0.91) | 0.98 (0.97–1.02) | **<0.001** |
| **BFM, kg** | 30.0 (24.8–36.5) | 22.6 (15.2–27.5) | **0.002** |
| **TFM, kg** | 15.2 ± 5.1 | 13.5 ± 5.1 | 0.238 |
| **USFF, %** | 14.3 (8.6–17.5) | 10.4 (8.3–15.7) | 0.280 |
| **FLI** | 57.4 (30.2–89.8) | 77.3 (54.7–84.5) | 0.166 |
| **FG, mg/dL** | 91.5 (86.0–105.5) | 116.0 (96.0–124.0) | **0.008** |
| **HbA1c, %** | 6.05 (5.90–6.70) | 6.60 (6.10–7.30) | 0.063 |
| **FI, μU/mL** | 11.6 (7.8–17.1) | 11.4 (6.3–15.8) | 0.599 |
| **HOMA-IR** | 3.04 (1.83–4.13) | 3.20 (1.81–5.93) | 0.880 |
| **HOMA-β** | 124.7 (83.4–179.2) | 66.1 (42.1–132.2) | **0.027** |
| **C-peptide, ng/mL** | 2.48 (2.19–3.59) | 3.00 (2.33–3.98) | 0.293 |
| **TG, mg/dL** | 132.5 (87.2–221.2) | 162.0 (100.0–226.0) | 0.312 |
| **TC, mg/dL** | 212.2 ± 43.7 | 195.2 ± 45.2 | 0.195 |
| **LDL-C, mg/dL** | 131.1 ± 35.0 | 124.6 ± 38.7 | 0.551 |
| **VLDL-C, mg/dL** | 26.5 (17.5–44.2) | 32.0 (20.0–45.0) | 0.327 |
| **HDL-C, mg/dL** | 46.2 (41.0–56.3) | 35.8 (29.9–41.7) | **<0.001** |
| **TG/HDL-C ratio** | 3.03 (1.53–5.17) | 4.23 (2.70–7.49) | **0.028** |
| **AST, U/L** | 22.5 (20.8–25.2) | 20.0 (18.8–26.0) | 0.466 |
| **ALT, U/L** | 27.0 (19.0–30.8) | 29.0 (19.0–40.0) | 0.401 |
| **GGT, U/L** | 19.0 (13.6–26.2) | 32.0 (28.0–43.0) | **<0.001** |
| **ALP, U/L** | 71.0 (59.2–89.2) | 70.0 (58.0–88.0) | 0.769 |
| **FGF-21, pg/mL** | 89.0 (87.2–90.6) | 88.9 (87.1–90.9) | 0.872 |
| **TIMP-1, ng/mL** | 151.0 ± 28.0 | 163.5 ± 36.7 | 0.203 |
| **HA, ng/mL** | 378.5 (301.7–484.7) | 523.4 (349.7–637.9) | 0.071 |
| **PIIINP, ng/mL** | 1.26 (0.72–1.34) | 1.24 (0.54–1.39) | 0.976 |
| **25-OH vitamin D, ng/mL** | 13.2 (10.2–21.2) | 10.7 (8.3–14.6) | 0.322 |
| **NFS** | −2.0 ± 1.0 | −2.0 ± 1.5 | 0.918 |
| **FIB-4** | 0.84 (0.55–1.03) | 0.71 (0.60–1.11) | 0.928 |
| **APRI** | 0.24 (0.21–0.29) | 0.24 (0.22–0.30) | 0.832 |
| **ELF score** | 9.07 (8.86–9.41) | 9.37 (8.98–9.69) | 0.160 |

Values are presented as mean ± SD for normally distributed variables and median (IQR) for non-normally distributed variables. Sex-stratified comparisons were performed within the MASLD-Dys subgroup only. Normally distributed variables were compared using Welch’s independent-samples t-test, and non-normally distributed variables were compared using the Mann–Whitney U test. No adjustment for multiple comparisons was applied because this analysis was exploratory. Bold p-values denote nominal statistical significance (p < 0.05). MASLD-Dys, metabolic dysfunction-associated steatotic liver disease with dysglycaemia; BMI, body mass index; WC, waist circumference; HC, hip circumference; WHR, waist-to-hip ratio; BFM, body fat mass; TFM, trunk fat mass; USFF, ultrasound-derived hepatic fat fraction; FLI, Fatty Liver Index; FG, fasting glucose; HbA1c, glycated haemoglobin A1c; FI, fasting insulin; HOMA-IR, Homeostatic Model Assessment of Insulin Resistance; HOMA-β, Homeostatic Model Assessment of β-cell Function; TG, triglycerides; TC, total cholesterol; LDL-C, low-density lipoprotein cholesterol; VLDL-C, very-low-density lipoprotein cholesterol; HDL-C, high-density lipoprotein cholesterol; AST, aspartate aminotransferase; ALT, alanine aminotransferase; GGT, gamma-glutamyl transferase; ALP, alkaline phosphatase; FGF-21, fibroblast growth factor 21; TIMP-1, tissue inhibitor of metalloproteinase-1; HA, hyaluronic acid; PIIINP, procollagen type III N-terminal propeptide; 25-OH vitamin D, 25-hydroxyvitamin D; NFS, NAFLD Fibrosis Score; FIB-4, Fibrosis-4 Index; APRI, Aspartate Aminotransferase to Platelet Ratio Index; ELF, Enhanced Liver Fibrosis.
